# Telegroup Visits for Caregivers and Patients With Dementia

**DOI:** 10.1093/geroni/igaa057.2284

**Published:** 2020-12-16

**Authors:** Stephen Thielke, Kris Fredrickson

**Affiliations:** 1 VA Puget Sound Health Care System, Seattle, Washington, United States; 2 Veterans Affairs, Seattle, Washington, United States

## Abstract

Group visits have shown promise for caregiver support and medical management of patients with dementia. In-person visits can be challenging to schedule and complete, particularly in rural areas where there are few specialists. We describe our experience with using telehealth modalities to hold group dementia visits. For the last four years, we have held telegroup appointments with Veterans with dementia and their caregivers. A geriatric psychiatrist and geriatric social worker appear from the main facility, and the Veterans and caregivers are at remote sites. Participants have actively engaged. They have expressed that the structure allows them to support and be supported by peers, and to have frequent contact with care providers. This has led to improved care metrics. The technology requirements are minimal. We discuss the advantages of this approach, including flexibility and maximizing use of specialist resources. We address challenges to scaling up such programs.

